# Section on Surgery

**Published:** 1866-07

**Authors:** A. C. Post, J. H. Burge

**Affiliations:** In the Chair; Secretary


SECTION ON SURGERY.

Dr. A. C. Post in the Chair; Dr. J. H. Burge, Secretary.

*First Session, May 1.*

At a meeting of the Surgical Section of the American Medical Association, held at the Dental College, corner of Hanover and Lombard streets, Baltimore, Md., May 1, 1866, at 3 o'clock P.M., Dr. Alfred C. Post, of New York, was elected Chairman, and Dr. J. H. Hobart Burge, Secretary.

There being no report of the papers which had been referred to this Section, a motion was made to adjourn, to meet again in the same place at 3 o'clock P.M., on Wednesday, 2d instant. Carried.

*Second Session, May 2.*

The Surgical Section met at 3 o'clock, pursuant to adjournment.

Dr. Post in the Chair.

Dr. J. S. Cohen, of Philadelphia, offered a paper by Dr. J. M. Boisnot of the same city, on Fracture of the Patella, and exhibited an apparatus for its treatment. On motion, the paper was referred to the Publication Committee.

Dr. J. S. Cohen read a paper on Vocal Paralysis, with Aphonia, cured by the application of stimuli and by the use of what he called vocal gymnastics. The paper was referred to the Publication Committee.

Dr. J. J. Woodward, U.S.A., chairman of Committee on Causes and Pathology of Pyæmia, presented a very elaborate paper. The Section listened for nearly an hour, when several members expressed their great interest in the communication, but owing to the shortness of the session it was voted to refer it at once to the Publication Committee, with the recommendation that it be published in full.

Dr. Woodward also presented some very beautiful photographs, illustrative of the success attained in micro-photography. In these specimens microscopes of very differing powers had been used.

Dr. A. C. Post, of New York, exhibited an original instrument for the bilateral operation of lithotomy.

Dr. J. C. Hutchinson, of Brooklyn, said he had used the instrument with satisfaction, and therefore moved that Dr. Post be requested to furnish a description for publication in the Transactions of this Association. Seconded and carried.

Dr. Post presented, also, a new instrument for making applications to the os uteri. It consists of a straight and rather slender pair of forceps, made short for convenience of carrying, but when in use elongated by a handle screwed thereupon. By a slide upon the forceps the blades are approximated and made to close firmly upon any object. The Doctor observed that it might be used either as a forceps to hold any substance which the operator might wish to apply, or to remove extraneous substances from the vagina.

Dr. Post also presented a small instrument to facilitate the introduction of insect pins for sutures; some of these pins being so delicate that without such guide they bend and become useless. The instrument consists of a sharp-pointed, slightly-curved, grooved needle—the groove being in its long axis. In using, the needle is to be introduced to the very position which the pin is intended to occupy. The pin is then guided by the groove, carried to its place, and held there while the needle is withdrawn in the backward direction.

Dr. H. R. Storer, of Mass., presented an instrument which he called "The Clamp Shield," designed by him to lessen the dangers of extirpation of the uterus by abdominal section, and read a paper on the subject, which was referred to the Committee on Publication.

